# First Genomic Evidence of Dual African Swine Fever Virus Infection: Case Report from Recent and Historical Outbreaks in Sardinia

**DOI:** 10.3390/v13112145

**Published:** 2021-10-25

**Authors:** Mariangela Stefania Fiori, Luca Ferretti, Matteo Floris, Federica Loi, Antonello Di Nardo, Anna Maria Sechi, Anna Ladu, Graziella Puggioni, Daria Sanna, Fabio Scarpa, Maria Luisa Sanna, Maria Paola Madrau, Claudia Torresi, Roberto Sirica, Eloisa Evangelista, Annalisa Oggiano, Silvia Dei Giudici

**Affiliations:** 1 Department of Animal Health, Istituto Zooprofilattico Sperimentale della Sardegna, 07100 Sassari, Italy; mariangela.fiori@izs-sardegna.it (M.S.F.); annamaria.sechi@izs-sardegna.it (A.M.S.); anna.ladu@izs-sardegna.it (A.L.); Graziella.puggioni@izs-sardegna.it (G.P.); marialuisa.sanna@izs-sardegna.it (M.L.S.); paola.madrau@izs-sardegna.it (M.P.M.); annalisa.oggiano@izs-sardegna.it (A.O.); silvia.deigiudici@izs-sardegna.it (S.D.G.); 2 Big Data Institute, Nuffield Department of Medicine, University of Oxford, Oxford OX1 4BH, UK; luca.ferretti@gmail.com; 3 Department of Biomedical Sciences, University of Sassari, 07100 Sassari, Italy; matteo.floris@gmail.com (M.F.); darsanna@uniss.it (D.S.); 4 Osservatorio Epidemiologico Veterinario Regionale, Istituto Zooprofilattico Sperimentale della Sardegna, 09125 Cagliari, Italy; 5 The Pirbright Institute, Ash Road, Pirbright, Woking GU24 0NF, UK; antonello.dinardo@pirbright.ac.uk; 6 Department of Veterinary Medicine, University of Sassari, 07100 Sassari, Italy; fscarpa@uniss.it; 7 Istituto Zooprofilattico Sperimentale dell’Umbria e delle Marche, 06126 Perugia, Italy; c.torresi@izsum.it; 8 Ames Polydiagnostic Group Center SRL, 80013 Napoli, Italy; roberto.sirica@centroames.it (R.S.); elo.evangelista@gmail.com (E.E.)

**Keywords:** African swine fever, dual infection, phylogenetic analysis, whole genome sequence, Sardinia

## Abstract

African swine fever virus (ASFV) is one of the pathogens of highest concern worldwide. Despite different virus lineages co-circulating in several areas, dual infections in the same animal have been rarely observed, suggesting that ASF superinfections are infrequent events. Here we present the first genome-wide detection and analysis of two intragenotype dual ASFV infections. The dual infections have been detected in a hunted wild boar and in a pig carcass, both infected by ASFV genotype I in Sardinia in 1984 and 2018, respectively. We characterize the genetic differences between the two sequences, their intra-host frequency, and their phylogenetic relationship among fully sequenced ASFV strains from Sardinia. Both dual infections involve pairs of closely related but different viruses that were circulating in Sardinia in the same period. The results imply that dual ASFV infections or similar ASFV strains are more common than expected, especially in ASF endemic areas, albeit difficult to detect.

## 1. Introduction

African swine fever (ASF) is an infectious, often lethal disease affecting suid species caused by the African swine fever virus (ASFV) [1]. The virus is the only member of the family Asfarviridae, characterized by a large, double-stranded DNA genome of around 180–190 kb that encodes for over 150 open reading frames [1]. The virus mainly infects monocyte and macrophages but is also able to infect dendritic cells [1,2]. ASF is considered the most serious animal disease [3], given the lack of licensed vaccines [4], its capacity of affecting different target populations [5] with consequent high morbidity and mortality, and its transboundary and transcontinental spread [6]. The first ASF cases were reported in Kenya in 1914 [7], from where it spread to the Iberian Peninsula [8] and subsequently to Eastern Europe [9]. Since 2018, the ASFV has overrun the Asian continent [10].

Dual infections (i.e., infections by multiple variants of the same pathogen) are not uncommon in viral infections and represent a necessary condition for recombination [11]. Specific disease conditions are mandatory for the occurrence of such events; in particular, the prevalence of the disease should be high enough that it is not unlikely for a host to be repeatedly infected by two different strains that co-circulate in the same area at the same time [12]. It is not surprising that this event seems to be very rare for ASF. In Mozambique between 1960 and 1994, the presence of two genetically distinct viruses circulating simultaneously during the same outbreak has been confirmed, but no evidence of coinfection was detected [13]. Despite extensive evidence of co-circulation of different genotypes of ASFV in the same group of animals, dual infections were not detected, possibly because of technical limitations during those years. To date, the only evidence of infection with different strains was reported by Mulumba-Mfumu [14] and occurred in the Democratic Republic of the Congo during 2010, where two different variants of the central variable region (CVR) of the B602L gene associated with two strains both belonging to *genotype I* were sequenced from two different tissues of the same pig. However, such evidence relies on a single hypervariable region in a single sample; hence, alternative explanations such as intra-host mutations or mislabeling of one of the samples cannot be excluded. Some authors also suggested recombination processes during coinfections in ticks [15,16].

Considering the specific epidemiological context that may prompt dual infections (i.e., co-circulation of different strains in the same area within the same host population and high disease prevalence), the Mediterranean island of Sardinia (Italy) could be considered a favorable land for such rare events to occur. Molecular studies revealed that Sardinian ASFV belongs to *genotype I* (vp72) [13,17]. Additional studies focusing on other regions of the genome (p54) have classified Sardinian isolates within *genotype Ia* [18]. Differences were observed in the B602L gene, which is involved in viral morphogenesis [19,20] allowing the differentiation of Sardinian isolates in two temporally related subgroups (X and III). Almost all of the strains isolated from 1990 onwards (subgroup X) showed the deletion of 12–13 tetramers [21] with respect to those isolated before 1990 (subgroup III). Likewise, Sanna in 2017 [22] reported an identical temporal subdivision of Sardinian ASF viruses into two subgroups. These groups, differing from the deletion of a six-amino-acid, repeat at the C-terminus of the CD2v protein encoded by the EP402R (CD2 homolog). From the characteristic of the strains isolated after 1990, the gene was detected as responsible for adsorption of erythrocytes around infected cells (haemadsorption) facilitating virus spread in the host [19]. Almost all of the Sardinian ASF viruses isolated after 1990 (modern strains) showed deletions in both the B602L and EP402R genes if compared to viruses isolated before 1990 (historical strains). The first two Whole Genome Sequences (WGS) of Sardinian ASFV were obtained in 2016 [23,24]. Three more recent studies [17,25,26] analyzed 73 ASFV Sardinian full genomes showing a remarkable genetic stability of the strains.

In this paper, we present the first genome-wide analysis of dual infection with viruses belonging to the same ASFV *genotype I*, detected in both a wild boar and a domestic pig in 1984 and 2018, respectively, at the time of the first and the last epidemic peaks of ASF in Sardinia. We characterized the nucleotide differences between the different sequences in the viral population, their frequency within each sample, and their phylogenetic origin among Sardinian sequences and presented these as case report.

### 1.1. Dual Infections, Superinfections, and Coinfections

We borrow the nomenclature from studies of other viruses, such as Human Immunodeficiency Virus (HIV) and herpesviruses, where multiple infections have been observed and analyzed in great detail [27,28,29,30,31]. *Dual infections* are characterized by the presence of multiple strains (usually two) infecting the same individual. They can be conceptually categorized into *coinfections*, if all the strains were transmitted during the same exposure event, and *superinfections,* if they correspond to different exposure possibly from different sources.

### 1.2. Sardinian Epidemiological Context

The virus was introduced to Sardinia in 1978, probably through infected food waste coming from the Iberian Peninsula [32,33]. Although the first Sardinian eradication plan started during the 1980s, several risk factors (i.e., epidemiological, environmental, and sociocultural) allowed the persistence of the disease in the island until 2019 [34,35,36,37,38]. After only six months from its first detection, ASFV recorded more than 12,000 dead pigs [32,39]. The difficulties in the implementation of disease control measures (i.e., killing of all animals infected or suspect of infection, movement and export ban) led to ~200 domestic pig outbreaks recorded during the first 5 years of the epidemic [33]. Consequently, the disease spread all over the island, mainly in the Nuoro province where ASFV infected the full spectrum of its target populations (i.e., domestic pigs, wild boar (WB), and free-ranging pigs). The initial epidemic peaks were recorded in 1979–1984 in domestic pigs and in 1985 in wild boar, finding the suitable conditions for endemicity for more than 42 years [21,36,38,39,40,41]. During the last ASF Eradication Program 2015–2018, a significant decrease in disease prevalence was observed, and some areas demonstrated being free from ASFV [42]. The main role of illegal free-ranging pigs in disease persistence and the secondary one of WB have been recently demonstrated [17,41]. Several municipalities of the Nuoro province have been historically defined as the main endemic area for ASFV circulation, where the virus has been maintained not only by the high animal density and contact rate [41] but more importantly by the lack of high biosecurity measures that afford the interactions between the three susceptible populations [36].

## 2. Materials and Methods

### 2.1. Ethic Statement, Sampling, and Virus Isolation

Fresh monocytes/macrophages from healthy crossbred pigs (*Sus scrofa domesticus*), 6 to 24 months old, were used for virus isolation from on-field samples. Healthy crossbred pigs were housed at the Experiment Station of Istituto Zooprofilattico Sperimentale (IZS) of Sardinia (‘Surigheddu’, Sassari, Italy). Animal husbandry and handling procedures were performed according to Legislative Decree n.26 of 4 March 2014 and in agreement with the Guide of Use of Laboratory Animals issued by the Italian Ministry of Health (available at: https://www.salute.gov.it/imgs/C_17_EventiStampa_355_intervisteRelatori_itemInterviste_1_fileAllegatoIntervista.pdf, access date: 20 January 2021), under the authorization No. 1232/2020-PR of 31/12/2020 by the Italian Ministry of Health. No animals were killed for the present work.

Virus isolation has been carried out since 1978 on samples collected during ASF case notification in Sardinia (i.e., domestic pig outbreaks on farms, hunted wild boar, or carcasses). The presence of infectious ASFV was assessed using the Malmquist test (haemoadsorption test) as described in the OIE Terrestrial Manual [42]. ASFV isolation was performed on homogenized spleen tissues from naturally infected animals. Tested samples were added to porcine two-day-old monocytes/macrophage monolayers, and cells were monitored daily for five days for hemadsorption effect. In the presence of haemadsorption, culture supernatant was collected and stored at −80 °C (ASF Virus Archive, IZS of Sardinia, Italy). For negative samples, the Malmquist test was repeated by adding culture supernatants into fresh monocytes/macrophages; after three negative results, the absence of live ASFV virus was declared [42].

In this study, we obtained the WGS of the historical strain NU1984 isolated from a wild boar sampled in Nuoro province (unknown municipality) in 1984 and of the strain LO2018 isolated in 2018 from a domestic pig carcass (lat 39.97506, long 9.66024, Lotzorai municipality, Nuoro province) [26]. Sequencing of these strains was performed within the remit of a research project aimed at obtaining the WGS of several Sardinian ASFV strains collected between 1978 and 2018 [26]. The analyses are still ongoing, but all strains were checked for dual infections.

### 2.2. DNA Extraction, Quantification, and Sequencing

Viral DNA was extracted from cell culture supernatant to perform the sequencing through the Illumina platform using a QIAmp UltraSens Virus Kit (Qiagen, Hilden, Germany) according to the manufacturer’s instructions. DNA quantification was performed using an Epoch microplate spectrophotometer (BioTek, Winooski, VT, USA) and a Qubit 2.0 Fluorometer (Thermo Fisher Scientific, Waltham, MA, USA) according to the manufacturer’s instructions.

Two different Next Generation Sequencing (NGS) Illumina protocols were used to obtain the complete genome sequences of the two ASF strains used in this study. The NU1984 viral DNA libraries were prepared using the Illumina Nextera XT DNA sample preparation protocol (Illumina, San Diego, CA, USA). The libraries of NU1984 were sequenced using the HiSeq 2500 platform (Illumina) generating paired-end reads 2 × 150 at the Center for Advanced Studies, Research and Development in Sardinia, Pula (CRS4) on 17 September 2018.

The libraries of the LO2018 virus were prepared using a Nextera DNA Flex Library Prep Kit (Illumina Inc., San Diego, CA, USA) and sequencing was carried out on the Novaseq 6000 (Illumina) generating paired-end reads 2 × 150 at AMES Group, Instrumental Polydiagnostic Center Srl Naples, Italy on 11 May 2019. The sequences of the B602L (bases 96322–97938) and the EP402R genes (bases 68,928–70,112) were confirmed by repeated Sanger sequencing using the primers and the methods described previously [22].

### 2.3. Bioinformatic Analysis

Genome data processing was performed using an in-house bioinformatic pipeline. The bcl2fastq program (https://support.illumina.com/sequencing/sequencing_software/bcl2fastq-conversion, access date: 10 March 2021) was used to convert BCL files generated by the sequencing systems to standard FASTQ file formats. Trim Galore (https://github.com/FelixKrueger/TrimGalore, access date: 10 March 2021) was used to quality trim the data and remove sequencing adaptors. The reads were then aligned to the pig reference genome (*Sus scrofa* 10.2) [43] using the bwa-mem algorithm [44]. Only reads mapping uniquely to the ASFV genome were retained and realigned using GEM [45]. Aligned bam files were sorted and indexed with SAMtools [46] and deduplicated with Picard tools (https://broadinstitute.github.io/picard, access date: 10 March 2021). To obtain high-quality variants, FreeBayes [47] was used to call variants for each sample using the KX354450 [24] sequence as reference genome (parameters: “--ploidy 1 -X -u -m 20 -q 20 -F 0.2”). WGSs were aligned using MAFFT v. 7.427 [48] and polymorphism positions were visually inspected using Jalview v. 2.10.3 B.1 software [49]. Bam files of both NU1984 and LO2018 were aligned with the KX354450 sequence and visually inspected with IGV v. 2.4.14 [50] to detect dual infections. The program *GC Content Calculator* (https://jamiemcgowan.ie/bioinf/gc.html, access date: 10 March 2021) was used to calculate the % G~C content. Genome annotation was performed using *GATU software* [51] using KX354450 as the reference genome.

### 2.4. Inference of Strain Frequency and Composition from Variant Frequencies

There are well-known approaches to detect dual infections from a collection of intra-host sequences [52]. However, when it is not possible to reconstruct such sequences due to short reads and low diversity, dual infections from two different strains can be detected by a different approach based on the frequency distribution of variants, as inferred from deep sequencing of short reads [53]. This approach is based on the observation of a large number of variants of similar, intermediate frequency. For NU1984 and LO2018, we selected all nucleotide variants with minor allele frequencies of >10% and covered by at least two reads, located in bases with minimum coverage 10. Variants were pre-called using SiNPle [54] with posterior probability >90%. To infer the frequencies of the two strains and their most likely sequences, we considered all possible frequencies of the two strains and all possible assignments of variants. We inferred the most likely using an ML approach, based on baseline frequencies and composition of all the Sardinian full genomes sequenced and published so far [17,23,24,25] (Table S1). For the selected variants, the likelihood was based on random sampling of both sets of variants from the initial set of Sardinian sequences and the binomial sampling of reads from the two sets of variants. More precisely, if a sequence composition *s* appears *n_s_* times among Sardinian sequences, with an additional pseudo-count of *n_s_ = 1* for combinations not present among Sardinian sequences, the likelihood is the product of *n_s_/Σ_s_,n_s_* and of the product among all variants of the binomial likelihood

(1) $$\left(\frac{c_{1}+c_{2}}{c_{1}}\right)f_{1}{}^{c_{1}}f_{2}{}^{c_{2}}$$

with $\;f_{1}\;$ and $f_{2}$ denoting the frequencies of the two strains in the viral population (which must satisfy $\;f_{1}+f_{2}=1$) and $c_{1},c_{2}$ the read counts. Confidence intervals were inferred from likelihood profiling and the Likelihood-Ratio test using Wilks’ theorem (1938) [55]. The whole analysis was implemented in R-software (Version 3.6.2, R-Foundation for Statistical Computing, Vienna, Austria). Once the two coinfecting virus variants within the NU1984 and LO2018 isolates were assembled by ML, the two haplotypes resulted in four different sequences named NU1984_major, NU1984_minor, LO2018_major, and LO2018_minor. The four reconstructed sequences and all the Sardinian ASFV whole genomes were then aligned to analyze differences between strains, their location, and their function. Their genome sequences were deposited in GenBank under the following accession number: MW647171/SRR15179098 (LO2018_major) and MW647172/ SRR15179098 (LO2018_minor); SRR13785534 (NU1984).

### 2.5. Phylogenetic Analysis

The ML tree was built from all the whole genome Sardinian strains sequenced so far [17,23,24,25] plus two outgroups (LI/1960 from Portugal and ES/1975 from Spain), and details are provided in Table S1. Sequences were aligned with *MAFFT* v. 7.427 [48]. The more likely assignment was defined based on AIC, and the inferred ML frequency for minor and major haplotypes are presented as frequency (%) and a 95% confidence interval (95% CI). The first and last 2000 bases of the genome were removed to avoid alignment artifacts. The ML tree was built using RAxML v. 8.2.12 [56] with 1000 bootstraps and GTR-CAT as the evolutionary model. The NJ tree was inferred using bioNJ* [57] from distances computed according to the TN93 model implemented in APE [58].

## 3. Results

### 3.1. Bioinformatic Analysis

We obtained the consensus genome sequence for samples LO2018 [26] and NU1984. Both sequences were deposited in the NCBI Short Read Archive (SRA) (accession numbers are reported in Table S1). The median coverage for NU1984 and LO2018 was estimated and reported in Table S2. The inverted terminal repeats (ITRs) that included KP86R, KP96L, DP93R, and DP86L genes were missed at both ends (this is probably linked to the difficulties in assembling reads due to low coverage of these regions). The comparison of LO2018 and NU1984 with the *genotype I* genome sequences (Table S1) available from the NCBI GenBank repository allowed us to observe point mutations, including insertions or deletions (indels), described in Table S3 and in Torresi et al. (2020) [25].

The genomic content of all known Sardinian sequences is very similar [25], and the same is true for NU1984 and LO2018. The sequence of the LO2018 strain was 181,758 bp long with GC content of 38.57%. Following annotation by *GATU* software, a total of 231 ORF in LO2018 were identified, with 165 protein-coding genes. The replacement of A > C base (27%) in position 107474 (relative to KX354450) causes a start codon loss in the URF 33 gene, resulting in the absence of this hypothetical protein; this finding was also observed in other Sardinian isolates (22,653, 103,917 and DE/2018 [17,25]).

### 3.2. Evidence for Dual Infection

**Figure 1:**
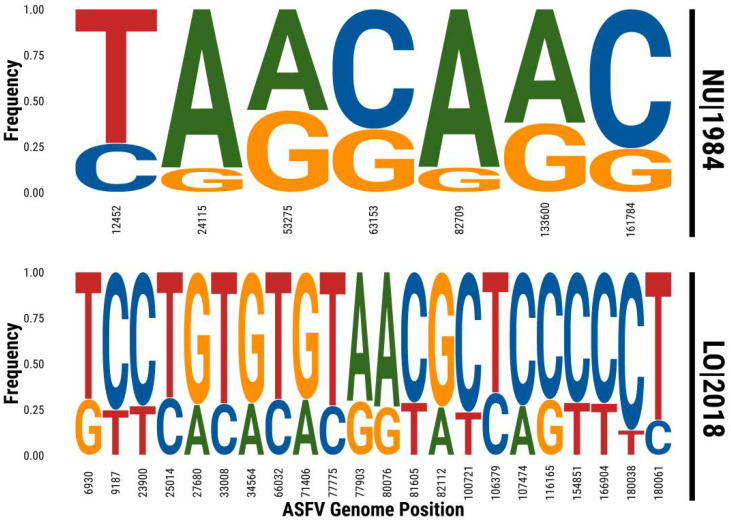
Sequence logo for intra-host polymorphisms. Each column represents the nucleotide content of a polymorphic site, with nucleotide size proportional to the relative frequencies among mapped reads. Genomic positions relative to the KX354450 sequence are reported below for each site.

We analyzed reads from deep sequencing of the samples NU1984 and LO2018, comparing them with 13 Sardinian genome sequences previously published [17,25] and reads from 58 further Sardinian ASFV strains collected between 1978 and2018 [26], scaffolded against the KX354450 reference genome. NU1984 and LO2018 are the only two samples showing a large number of intra-host nucleotide polymorphisms, illustrated in Figure 1 and Table 1.

In the NU1984 sample, we find a set of seven intra-host variants at intermediate frequencies between 13% and 47%, with an average frequency of ~28% (SD = 12%). In the LO2018 sample, we find 22 variants with a range of minor allele frequencies between 23% and 34%. Given the low mutation rate and very low intra-host variability of ASFV [5], such variants are unlikely to be the result of intra-host mutations. The natural alternative is that they originated from multiple variants infecting the same animal at some point; therefore, these ASFV infections are excellent candidates for dual infections. For NU1984, the frequencies look more dispersed than expected (overall SD is about 2.3 times the one expected under binomial sampling of reads), but the modest coverage of the positions where the variants are located (11 reads only for some variants) and possibly the additional noise due to viral DNA extraction and amplification are likely explanations for this over-dispersion in frequencies. The variants and their allele counts are reported in Table 1. No further evidence suggestive of coinfections was found among the other Sardinian strains analyzed. Known variable regions in B602L and EP402R of NU1984 and LO2018 samples were also sequenced several times. Repeated Sanger sequencing of NU1984 for the B602L region provided a consistent result, finding a deletion in all seven replicates analyzed. However, sequences of the EP402R region obtained by Sanger sequencing showed clear genetic differences: the same deletion that is present among consensus sequences is found in approximately 70% (21 out of 30) of them, 3 out of 30 replicates clearly showed no deletion, and 6 out of 30 replicated gave inconclusive results due to the presence of several double peaks within the deletion region. Since it is very unlikely that intra-host processes would generate precisely the same deletion found in sequences of other viruses isolated from the same epidemic, this is clear evidence of a dual infection at intermediate frequencies. On the other hand, repeated LO2018 Sanger sequencing for B602L and EP402R regions consistently showed deletions in all sequences for both regions.

### 3.3. Inference of Strain Frequency and Composition from Variant Frequencies

The Maximum Likelihood (ML) analysis for NU1984 confirms that the most likely sequence contains all major alleles TAACAAC and all minor alleles CGGGGGG. This is the most likely assignment, since the next most likely one (a switch of the major and minor alleles in position 12,452) is strongly penalized by the difference in the Akaike Information Criterion (AIC) score of 27.4. The inferred ML frequency for the minor haplotype is 31% (95% CI: 28–35%) and therefore 69% for the major haplotype (95% CI: 65–72%). The same analysis for LO2018 indicates that the most likely sequence containing all major alleles was TCCTGTGTGTAACGCTCCCCCT and the corresponding minor alleles was GTTCACACACGGTATCAGTTTC. This is by far the most likely assignment given that the difference in the AIC score of 324.4 strongly disfavors the next most likely one (a switch of the major and minor alleles in the last position 180,061). The inferred ML frequency for the minor haplotype is 28% (95% CI: 27–29%) and therefore 72% for the major haplotype (95% CI: 71–73%).

### 3.4. Phylogenetic Analysis

**Figure 2:**
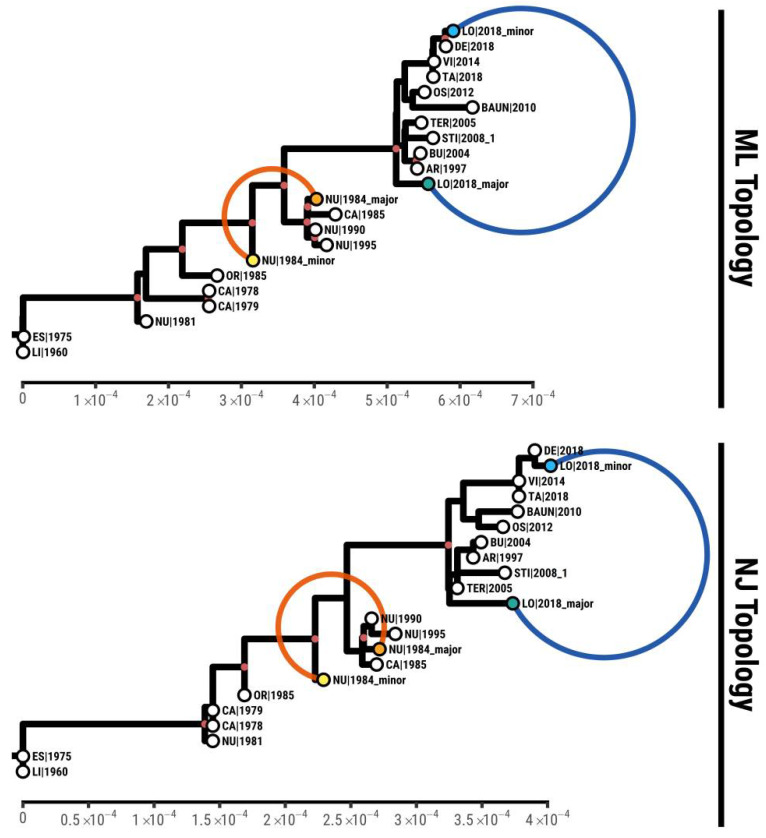
Maximum Likelihood and Neighbor Joining phylogenies reconstructed from Sardinian ASFV genotype I sequences. Tips characterizing the dual infections are represented in blue (LO|2018) and orange (NU|1984). Nodes with supported bootstrap values of >90 are colored in red.

From the comparison of the polymorphisms in NU1984 and the consensus bases in other Sardinian strains (Table S1), it is evident that the major haplotype is very similar to those sequences sampled after the mid-1980s but with two exclusive variants, while the minor one is more closely related to those viruses circulating during the early years of the Sardinian epidemic. We reconstructed the phylogenetic tree including the sequences inferred for major and minor strains in these dual infections found in both samples. The Neighbor Joining (NJ) tree and ML tree in Figure 2 show how both sequences in NU1984 are consistently grouped among other sequences sampled between 1985 and1995.

The major and minor alleles of LO2018 are spaced out in both the NJ and ML trees: the minor groups with other sequences from 2014 to2018, while the major is assigned sister to the clade that includes isolates from 1997 to 2008, with six exclusive variants. While the most frequent haplotype of NU1984 in the dual infection is assigned within a clade of sequences from 1985 to1995 (which correspond to those samples collected after the 1984 epidemic peak), the less frequent haplotype corresponds to a virus that can be considered an evolutionary intermediate between the viruses circulating pre-1984 and those occurring from 1985 to 1995. Focusing on LO2018, the least frequent haplotype is a close relative of known sequences from the same year and geographical area, while the most frequent one is a basal lineage for the later phase of the Sardinian epidemic. The consistent phylogenetic placement of the two coinfecting viruses is further confirmation of the correct inference of their sequences and that these intra-host polymorphisms originate from dual infections rather than evolution within hosts.

### 3.5. Genomic Differences between Coinfecting Strains

In this study we confirm the genome differences between Sardinian strains previously detected [17,25,26], and we further report newly identified point mutations. Genetic differences between Sardinian viruses with the strains analyzed in this study are summarized in Table S3 (NU1984 major and minor), S4, and S5 (LO2018 major and minor). A total of 14 mutations in 13 genes and 1 hypothetical protein or URF (unassigned reading frame) were found (8 synonymous mutations and 6 non-synonymous mutations). As discussed, only seven nucleotide variants were observed between the two coinfecting viruses (NU1984_major and NU1984_minor), corresponding to a very low divergence of about 4 × 10^−5^ mutations per base. Two variants exclusive to NU1984_major were observed, located in the region coding for the genes EP1242L (involved in nucleic acid synthesis) and in the gene D250R (coding for the mRNA-decapping protein g5R). The replacement of the G > C base (65%) in position 63153 (relative to KX354450), within the EP1242L gene, is a silent point mutation. The replacement of the A > G base (62%) in position 133,600 produced an amino acid mutation at position 157 in the D250R gene, corresponding to a E > K substitution. This mutation is located within the Nudix hydrolase signature domain, which has been shown to regulate the virulence of ASFV by altering or inhibiting the expression of host proteins through the degradation of mRNA by means of decapping the methylated cap attached to mRNA [59,60]. A BLAST analysis revealed that this non-synonymous nucleotide change is present only in NU1984_major respective to all the D250R gene sequences present in GenBank. The assignment of the deletion in the EP402R region is further evidenced in the tree topologies presented above. In fact, the deleted allele is consistently present in the cluster of sequences phylogenetically associated to the major strain. Hence, the most parsimonious explanation is that the major strain presents only the deletion in B602L, while the minor strain presents deletions in both EP402R and B602L regions. Regarding the sample LO2018, a total of 22 variants relative to KX354450 were observed, but only 8 were exclusive (3 non-synonymous and 5 synonymous mutations, Table S3). These are located within the genes MGF110-1L and MGF505-5R (MGF families), EP84R (coding for a transmembrane protein), C717R (belonging to a PK-like protein superfamily), M1249L (coding for a capsid component) [28], and C122R (coding for a structural component of the virus particle) [29]. Two mutations (one non-synonymous and one synonymous) were found within the gene DP96R, involved in virus virulence [61]. Further, two non-synonymous and two synonymous mutations, already described [25,26], are located within the genes MGF110-13L_1 (MGF 110 family), Lis117, URF11, and QP509L and are restricted to both LO2018_minor and DE/2018 (GenBank MT932579) [17]. The deletions found in the CD2 and B602L regions are present in both major and minor strains.

## 4. Discussion

In this study, we analyzed two Sardinian ASF viruses, which were collected from the most endemic area of Sardinia and were isolated from a wild boar and a domestic pig during the 1984 and 2018 endemic peaks, respectively. The analysis of NU1984 and LO2018 [26] WGSs revealed the presence of two different viral strains in each sample that we named NU1984_major and NU1984_minor and LO2018_major and LO2018_minor, respectively. Dual infections require a superinfection event, which happens when two or more viral strains are transmitted to the same animal at different times [12]. Our results suggest that even if ASFV superinfections are very difficult to detect, these could be more common than expected, at least in the ASF endemic context of Sardinia characterized by several epidemic peaks. In fact, most superinfections will involve viruses with almost identical sequences, and the two viruses are unlikely to be found in similar proportions unless the second infection occurs shortly after the first one. This observation has two important bases. First, there is a high incidence and prevalence of the disease in the central areas of Sardinia during the 1984 and 2018 peaks; otherwise, occurrence of multiple transmissions to the same animal in a short period of time would not have been possible. Second, during the same years, there was abundant co-circulation of multiple viral strains within the local host populations; otherwise, such dual infections could not originate. There is no reason to believe that these findings would be unique to ASFV genotype I or to the Sardinian epidemic. We expect that coinfections could be found in other ASF endemic areas having a similar epidemiological context. The presence of dual infections is a precondition for recombination; hence, the evidence in this paper implies that recombination between ASFV strains is likely to occur and supports the recent phylogenetic evidence for recombination as a relevant evolutionary force acting on ASFV genomes. Note that the presence of such dual infections suggests that either superinfection exclusion does not occur in ASFV infections, despite the similarity of the coinfecting strains, or that the occurrence of these dual infections may have also been facilitated by functional differences among the coinfecting haplotypes, e.g., because their genetic variation could have triggered differences in virulence or intra-host growth. Some of these variants are found exclusively in these samples, for example, in NU1984, the silent mutation for EP1242L gene encoding RNA polymerase and the amino acid substitution in D250R gene encoding the mRNA-decapping protein g5R. The latter mutation occurs within the Nudix hydrolases signature domain, which has been shown to play a role in the virulence of ASFV by altering or inhibiting the expression of host proteins through the degradation of mRNA by means of decapping the methylated cap attached to mRNA [59]. This non-synonymous change was unique to the NU1984_major haplotype. The sample LO2018 contained several variants that could cause a functional difference between haplotypes, located in MGF505-5R (MGF 505 family), C717R, and DP96R, as well as synonymous silent mutation for MGF110-1L (MGF 110 family), Ep84R, M1249, C122R, QP509L, and DP96R genes. LO2018_minor contained the variant between haplotypes, located in MGF110-13L_1 and Lis117, as well as synonymous silent mutation for URF11 and QP509L present only in DE/2018 (GenBank accession number MT932579) isolated in 2018 [17]. A recent study [62] suggests that a mutant of QP509L, the DEAH box ATP-dependent RNA helicase gene, which is essential for viral replication, can be a good candidate for a live attenuated vaccine by not producing progeny and inducing a protective immune response. As described in previous studies, the ASF prevalence detected within illegal free-ranging pigs in Sardinia was much higher than the prevalence detected in the other two ASF host populations (wild boar and domestic pigs) [35,41]. The increasing probability of detecting coinfection in an epidemiological context of high disease prevalence could partially explain this finding in LO2018. Given the distant location of the minor and major strains in the ML tree, further investigations are ongoing to evaluate the variability of the ASFV population within the closed ecosystem of Sardinia. In conclusion, this is the first report of the presence of ASFV dual infections based on clear genomic evidence. Given the technical difficulties in detecting such infections, our finding of two different dual infections out of less than a hundred samples strongly suggests that dual ASFV infections are relatively common events in Sardinia and linked to areas of high disease prevalence and frequent co-circulation of multiple strains.

## Figures and Tables

**Table 1:** Position with respect to the reference sequence KX354450, major and minor allele and their read counts for all intermediate variants, in NU1984 and LO2018 samples.

Sample	Position in Reference	Major Variant	Minor Variant	Major Read Count	Minor Read Count	Major Frequency	Minor Frequency
NU1984	12452	T	C	11	4	0.73	0.27
	24115	A	G	13	2	0.87	0.13
	53275	A	G	52	42	0.55	0.45
	63153	C	G	144	76	0.65	0.35
	82709	A	G	57	9	0.86	0.14
	133600	A	G	28	17	0.62	0.38
	161784	C	G	70	22	0.76	0.24
LO2018	6930	T	G	200	88	0.69	0.31
	9187	C	T	257	84	0.75	0.25
	23900	C	T	275	101	0.73	0.27
	25014	T	C	228	104	0.69	0.31
	27680	G	A	259	98	0.73	0.27
	33008	T	C	227	89	0.72	0.28
	34564	G	A	228	90	0.72	0.28
	66032	T	C	233	102	0.70	0.30
	71406	G	A	220	96	0.70	0.30
	77775	T	C	263	97	0.73	0.27
	77903	A	G	244	102	0.71	0.29
	80076	A	G	243	87	0.74	0.26
	81605	C	T	217	87	0.71	0.29
	82112	G	A	239	85	0.74	0.26
	100721	C	T	270	83	0.76	0.24
	106379	T	C	240	124	0.66	0.34
	107474	C	A	230	85	0.73	0.27
	116165	C	G	203	89	0.70	0.30
	154851	C	T	294	118	0.71	0.29
	166904	C	T	262	103	0.72	0.28
	180038	C	T	102	16	0.86	0.14
	180061	T	C	110	26	0.81	0.19

## Data Availability

Not applicable.

## Supplementary Materials

The following are available online at https://www.mdpi.com/article/10.3390/v13112145/s1, Table S1: African swine fever virus (ASFV) whole-genome sequences available from public databases; Table S2: Sample coverage; Table S3: Point mutation.
