# Supplementary material for: First Genomic Evidence of Dual African Swine Fever Virus Infection: Case Report from Recent and Historical Outbreaks in Sardinia
Source: Viruses. 2021 Oct 25;13(11):2145. doi: 10.3390/v13112145 (PMC8618892; doi:10.3390/v13112145)
Supplement: Supplementary file 1 [file viruses-13-02145-s001.zip › viruses-1407570-supplementary.pdf]

Table S1. African swine fever virus (ASFV) whole-genome sequences available from public databases

Number	Accession Number	Asfv Isolate/ Beast code	Country of Origin	Province/Town	Submission Date	Collection Date	Host	P72 Genotype	Methods	Reference
1	KM262844	L60	Portugal	Spain	2014	1960	Domestic pig	I	Sanger sequencing	De Villiers et al.. 2016
2	FN557520	E75	Spain	Lisbona	2009	1975	Domestic pig	I	Sanger sequencing	Portugal et al. 2015
3	MN270969	CA|1978	Italy (Sardinia)	Cagliari	2020	1978	Domestic pig	I	NextSeq Illumina, Sanger sequencing	Torresi et al.2020
4	MN270970	CA|1979	Italy (Sardinia)	Cagliari	2020	1979	Domestic pig	I	NextSeq Illumina, Sanger sequencing	Torresi et al.2020
5	MN270971	NU|1981	Italy (Sardinia)	Nuoro	2020	1981	Domestic pig	I	NextSeq Illumina, Sanger sequencing	Torresi et al.2020
6	SRR13785534	NU|1984	Italy (Sardinia)	Nuoro	2020	1984	Wild Boar	I	NextSeq Illumina, Sanger sequencing	This study
7	MN270972	OR|1985	Italy (Sardinia)	Oristano	2020	1985	Domestic pig	I	NextSeq Illumina, Sanger sequencing	Torresi et al.2020
8	MN270973	CA|1985	Italy (Sardinia)	Cagliari	2020	1985	Domestic pig	I	NextSeq Illumina, Sanger sequencing	Torresi et al.2020
9	MN270974	NU|1990	Italy (Sardinia)	Nuoro	2020	1990	Domestic pig	I	NextSeq Illumina, Sanger sequencing	Torresi et al.2020
10	MN270975	NU|1995	Italy (Sardinia)	Nuoro	2020	1995	Domestic pig	I	NextSeq Illumina, Sanger sequencing	Torresi et al.2020
11	MN270976	AR|1997	Italy (Sardinia)	Nuoro/Aritzo	2020	1997	Domestic pig	I	NextSeq Illumina, Sanger sequencing	Torresi et al.2020
12	MN270977	BU|2004	Italy (Sardinia)	Sassari/Bultei	2020	2004	Domestic pig	I	NextSeq Illumina, Sanger sequencing	Torresi et al.2020
13	MN270978	TER|2005	Italy (Sardinia)	Sassari/Tergu	2020	2006	Wild Boar	I	NextSeq Illumina, Sanger sequencing	Torresi et al.2020
14	KX354450	SS|2008	Italy (Sardinia)	Sassari/Stintino	2016	2008	Domestic pig	I	Illumina MiSeq; PacBio	Granberg et al., 2016
15	KM102979	BAU|2010	Italy (Sardinia)	Nuoro/Baunei	2014	2010	Domestic pig	I	Illumina HiScanSQ, Sanger sequencing	Bacciu et al., 2016
16	MN280979	OS|2012	Italy (Sardinia)	Sassari/Oschiri	2020	2012	Domestic pig	I	NextSeq Illumina, Sanger sequencing	Torresi et al.2020
17	MN280980	VI|2014	Italy (Sardinia)	Cagliari/Villanovatulo	2020	2014	Domestic pig	I	NextSeq Illumina, Sanger sequencing	Torresi et al.2020
18	MT932578	TA|2018	Italy (Sardinia)	Talana/Nuoro	2020	2018	Domestic pig (free-ranging)	I	NextSeq Illumina, Sanger sequencing	Franzoni et. al. 2020
19	MT932579	DE|2018	Italy (Sardinia)	Desulo/Nuoro	2020	2018	Domestic pig (free-ranging)	I	NextSeq Illumina, Sanger sequencing	Franzoni et. al. 2020
20	MW647171/ SRR15179098	LO|2018_major	Italy (Sardinia)	Lotzorai/Nuoro	2020	2018	Domestic pig (free-ranging)	I	NextSeq Illumina, Sanger sequencing	This study

21	MW647172/ SRR15179098	LO|2018_minor	Italy (Sardinia)	Lotzorai/Nuoro	2020	2018	Domestic pig (free-ranging)	I	NextSeq Illumina, Sanger sequencing	This study

Table S2. Sample coverage

Sample ID	Diagnostic centre-NGS	Genome Territory	Mean Coverage	SD Coverage	Median Coverage
NU84WB	A	184638	69,42	57,98	53
54684/LO_2018	B	184638	243,52	33,23	250

A = AMES Group NAPOLI; B = CRS4 Pula

Table S3. Point mutation

Mutation n°	GENE	Genome Position	Ref	Alt Nu84 major	NU84WB-Minor	Alt 54684/LO 2018 major	54684/LO 2018Minor	Aa_chg +	Mutation	CA1978 /MN270969	CA1979 /MN270970	NU1981 /MN270971	OR1985 /MN270972	CA1985 /MN270973	NU1990 / MN270974	NU1995 /MN270975	AR|1997M N270976	BU|2004/M N270977	TER|2005/MN270978	BAU|2010/KM102979	OS|2012 /MN280979	VI|2014 /MN280980	TA|2018/MT932578	DE|2018/MT932579	Predicted features
1	MGF110-1L	6930	G	G	G	T	G(31%)	Silente	Synonymous mutations	G	G	G	G	G	G	G	G	G	G	G	G	G	G	G	MGF 110 family _Transmembrane helix (121-139);(145-165)
2	MGF 110-13L_1	9187	C	C	C	C	T(25%)	N132S	Non Synonymous mutations	C	C	C	C	C	C	C	C	C	C	C	C	C	C	T	MGF 110 family _Transmembrane helix (21-41);(47-66)
3	LIS117	9187	C	C	C	C	T(25%)	N84S	Non Synonymous mutations	C	C	C	C	C	C	C	C	C	C	C	C	C	C	T	N.A
4	URF11	27680	G	G	G	G	A(27%)	Silente	Synonymous mutations	G	G	G	G	G	G	G	G	G	G	G	G	G	G	A	hypothetical protein
5	MGF505-5R	33008	C	C	C	T	C(28%)	Y34H	Non Synonymous mutations	C	C	C	C	C	C	C	C	C	C	C	C	C	C	C	mgf 505 family
6	EP1242L	63153	G	C	G 35%	G	G	Silente	Synonymous mutations	G	G	G	G	G	G	G	G	G	G	G	G	G	G	G	RNA polymerase subunit 2/nucleoidd/transcriptiond

																									(993-1005)
7	EP84R	66032	C	C	C	T	C(30%)	Silente	Synonymous mutations	C	C	C	C	C	C	C	C	C	C	C	C	C	C	C	Transmembrane protein EP84R
8	M1249L	71406	A	A	A	G	A(30%)	Silente	Synonymous mutations	A	A	A	A	A	A	A	A	A	A	A	A	A	A	A	Uncharacterized protein (Capsid component)
9	C717R	77775	C	C	C	T	C(27%)	W21R	Non Synonymous mutations	C	C	C	C	C	C	C	C	C	C	C	C	C	C	C	Uncharacterized protein_Protein kinase-like (PK-like) superfamily
10	C122R	80076	G	G	G	A	G(26%)	Silente	Synonymous mutations	G	G	G	G	G	G	G	G	G	G	G	G	G	G	G	Uncharacterized protein (structural component of the virus particle)
11	D250R	133600	G	A	G 38%	G	G	E157K	Non Synonymous mutations	G	G	G	G	G	G	G	G	G	G	G	G	G	G	G	mRNA-decapping protein g5R-Nudix hydrolase (97-239)_Nudix box (132-153)
12	QP509L	154851	C	C	C	C	T(29%)	Silente	Synonymous mutations	C	C	C	C	C	C	C	C	C	C	C	C	C	C	T	Helicase ATP-binding (110-262) motif DEAH box (215-218)
13	DP96R	180038	C	C	C	C	T(14%)	S32P	Non Synonymous mutations	C	C	C	C	C	C	C	C	C	C	C	C	C	C	C	Uncharacterized protein (gene involved in virus virulence)
14	DP96R	180061	T	T	T	T	C(19%)	Silente	Synonymous mutations	T	T	T	T	T	T	T	T	T	T	T	T	T	T	T	Uncharacterized protein (gene involved in virus virulence)

Table S4. Genetic differences between Sardinian viruses with LO2018 Major allele

Gene Name	Product	Start	Stop	+/-	Size	Genetype	Score	% Identity
KP93L	pKP93L	71	160	-	90	GENE	65.4698	100

URF1	hypothetical protein	178	546	-	369	GENE	249.98	100
MGF 360-1L	pMGF 360-1L	655	1260	-	606	GENE	417.927	100
MGF 360-2L	pMGF 360-2L	1344	2432	-	1089	GENE	743.036	100
KP177R	pKP177R	2582	3115	+	534	GENE	370.548	100
L83L	pL83L	3253	3504	-	252	GENE	173.711	100
L60L	pL60L	3599	3802	-	204	GENE	130.568	100
MGF 360-3L	pMGF 360-3L	3987	4790	-	804	GENE	544.273	100
URF2	hypothetical protein	4729	5058	-	330	GENE	228.794	100
MGF 110-1L	pMGF 110-1L	5285	6097	-	813	GENE	564.688	100
URF3	hypothetical protein	5774	6070	+	297	GENE	199.519	100
MGF 110-13L	pMGF 110-13L	6260	6730	-	471	GENE	330.487	100
MGF 110-11L	pMGF 110-11L	6700	7065	-	366	GENE	253.447	100
MGF 110-12L	pMGF 110-12L	7254	7565	-	312	GENE	209.92	100
MGF 110-13L_1	pMGF 110-13L	7643	8140	-	498	GENE	337.806	99.4
LIS117	pLIS117	7643	7996	-	354	GENE	240.736	99.1
MGF 110-14L	pMGF 110-14L	8019	8468	-	450	GENE	313.153	100
MGF 360-4L	pMGF 360-4L	8792	9787	-	996	GENE	682.945	100
MGF 360-6L	pMGF 360-6L	10612	11739	-	1128	GENE	772.696	100
X69R	pX69R	12623	12832	+	210	GENE	140.584	100
MGF 300-1L	pMGF 300-1L	13019	13825	-	807	GENE	555.829	100
X64R	pX64R	14770	14964	+	195	GENE	132.88	100
MGF 300-2R	pMGF 300-2R	15545	16027	+	483	GENE	321.242	100
URF4	hypothetical protein	15919	16113	-	195	GENE	130.954	100
MGF 300-4L	pMGF 300-4L	16118	17104	-	987	GENE	681.019	100
MGF 300-3L	pMGF 300-3L	16118	16432	-	315	GENE	218.009	100
MGF 360-8L	pMGF 360-8L	17222	18181	-	960	GENE	665.226	100
URF5	hypothetical protein	18116	18373	+	258	GENE	172.94	100
MGF 360-9L	pMGF 360-9L	18343	19395	-	1053	GENE	733.406	100
MGF 360-10L	pMGF 360-10L	19503	20573	-	1071	GENE	731.865	100
MGF 360-11L	pMGF 360-11L	20599	21660	-	1062	GENE	733.791	100
URF6	hypothetical protein	20734	21039	+	306	GENE	203.756	100

URF7	hypothetical protein	21152	21400	+	249	GENE	154.451	100
MGF 505-1R	pMGF 505-1R	21867	23462	+	1596	GENE	1087.79	100
URF8	hypothetical protein	22742	22957	+	216	GENE	149.443	100
URF9	hypothetical protein	23043	23234	-	192	GENE	126.716	100
MGF 360-12L	pMGF 360-12L	23512	24564	-	1053	GENE	721.079	100
URF10	hypothetical protein	23978	24196	+	219	GENE	139.043	100
MGF 360-13L	pMGF 360-13L	24736	25797	-	1062	GENE	722.235	100
URF11	hypothetical protein	25969	26253	-	285	GENE	194.897	100
URF12	hypothetical protein	26281	26490	+	210	GENE	139.428	98.6
MGF 360-14L	MGF 360-14L	25969	27042	-	1074	GENE	495.352	66.4
MGF 505-2R	pMGF 505-2R	27248	28828	+	1581	GENE	1073.92	100
URF13	hypothetical protein	27808	27996	-	189	GENE	124.02	100
MGF 505-3R	pMGF 505-3R	28915	29757	+	843	GENE	576.244	100
URF14	hypothetical protein	29364	29558	-	195	GENE	131.724	100
MGF 505-4R	pMGF 505-4R	29922	31439	+	1518	GENE	1031.55	100
MGF 505-5R	pMGF 505-5R	31468	32964	+	1497	GENE	1013.83	100
MGF 505-6R	pMGF 505-6R	33162	34748	+	1587	GENE	1077.39	100
MGF 505-7R	pMGF 505-7R	34869	36275	+	1407	GENE	960.288	100
URF15	hypothetical protein	35895	36248	-	354	GENE	238.81	100
MGF 505-9R	pMGF 505-9R	36763	38283	+	1521	GENE	1045.42	100
MGF 505-10R	pMGF 505-10R	38607	40235	+	1629	GENE	1115.52	100
A224L	pA224L	40351	41025	-	675	GENE	467.233	100
A104R	pA104R	41387	41701	+	315	GENE	208.764	100
A240L	pA240L	41698	42420	-	723	GENE	495.352	100
A118R	pA118R	42332	42688	+	357	GENE	239.195	100
A151R	pA151R	42718	43173	+	456	GENE	313.538	100
MGF 360-15R	pMGF 360-15R	43518	44348	+	831	GENE	576.244	100
A238L	pA238L	44554	45270	-	717	GENE	495.352	100
URF16	hypothetical protein	45101	45313	+	213	GENE	137.887	100
URF17	hypothetical protein	45208	45420	+	213	GENE	142.895	100
URF18	hypothetical protein	45240	45449	-	210	GENE	141.739	100

A859L	pA859L	45310	47889	-	2580	GENE	1795.4	100
A179L	A179L	47891	48430	-	540	GENE	370.933	100
A137R	pA137R	48674	49087	+	414	GENE	284.648	100
F317L	pF317L	49120	50073	-	954	GENE	660.988	100
F334L	pF334L	50098	51102	-	1005	GENE	693.73	100
F778R	F778R	51123	53459	+	2337	GENE	1627.45	100
URF19	hypothetical protein	53346	53540	-	195	GENE	129.028	100
F165R	pF165R	53492	53989	+	498	GENE	344.739	100
F1055L	pF1055L	53976	57143	-	3168	GENE	2199.86	100
K205R	pK205R	57312	57929	+	618	GENE	420.624	100
BA71V-K78R	pK78R	58018	58254	+	237	GENE	152.525	100
K196R	pK196R	58251	58841	+	591	GENE	407.142	100
K145R	pK145R	58857	59294	+	438	GENE	292.738	100
K421R	pK421R	59327	60592	+	1266	GENE	869.381	100
EP1242L	pEP1242L	60611	64339	-	3729	GENE	2596.62	100
URF20	hypothetical protein	64037	64360	+	324	GENE	218.394	100
EP84R	EP84R	64418	64672	+	255	GENE	159.844	100
EP424R	pEP424R	64708	65982	+	1275	GENE	881.322	100
EP152R	pEP152R	66465	66923	+	459	GENE	317.005	100
EP153R	pEP153R	66926	67387	+	462	GENE	302.368	100
EP402R	pEP402R	67487	68671	+	1185	GENE	758.444	100
URF21	hypothetical protein	68211	68657	+	447	GENE	269.24	100
EP364R	pEP364R	68780	69874	+	1095	GENE	758.444	99.7
M1249L	pM1249L	69938	73261	-	3324	GENE	2308.49	100
M448R	pM448R	73727	75073	+	1347	GENE	936.406	100
C129R	pC129R	75160	75549	+	390	GENE	267.314	100
C84L	pC84L	75596	75850	-	255	GENE	168.703	100
C717R	pC717R	76274	78427	+	2154	GENE	1486.86	99.9
URF22	hypothetical protein	78411	78644	-	234	GENE	154.836	98.7
C122R	pC122R	78414	78731	+	318	GENE	217.238	100
C257L	pC257L	79130	79903	-	774	GENE	533.872	100

C475L	pC475L	79882	81309	-	1428	GENE	982.245	100
C315R	pC315R	81362	82309	+	948	GENE	649.432	100
C147L	pC147L	82377	82820	-	444	GENE	296.59	100
URF23	hypothetical protein	82453	83205	+	753	GENE	503.827	100
URF24	hypothetical protein	82837	83205	+	369	GENE	255.758	100
C62L	pC62L	82980	83168	-	189	GENE	124.79	100
C962R	pC962R	83250	86138	+	2889	GENE	2007.26	100
C962R_1	pC962R_1	84129	86138	+	2010	GENE	1403.27	100
URF25	hypothetical protein	86089	86247	-	159	GENE	105.531	100
URF26	hypothetical protein	86272	86487	-	216	GENE	147.132	100
B962L	pB962L	86276	89164	-	2889	GENE	2001.48	100
B119L	pB119L	89124	89483	-	360	GENE	247.669	100
URF27	hypothetical protein	89389	89658	+	270	GENE	182.185	100
B318L	pB318L	89464	90420	-	957	GENE	664.455	100
B438L	pB438L	90387	91703	-	1317	GENE	905.975	100
B169L	pB169L	91716	92225	-	510	GENE	340.117	100
B475L	pB475L	92242	93669	-	1428	GENE	958.362	100
B354L	pB354L	93806	94870	-	1065	GENE	736.102	100
B602L	pB602L	94881	96497	-	1617	GENE	1116.68	100
URF28	hypothetical protein	95827	96033	+	207	GENE	123.635	100
URF29	hypothetical protein	96311	96520	-	210	GENE	139.813	100
B385R	pB385R	96552	97709	+	1158	GENE	801.201	100
B646L	pB646L	97807	99747	-	1941	GENE	1347.41	100
B125R	pB125R	99787	100164	+	378	GENE	255.373	100
B117L	pB117L	100137	100490	-	354	GENE	244.202	100
B407L	pB407L	100497	101720	-	1224	GENE	837.41	100
B175L	pB175L	101748	102275	-	528	GENE	365.54	100
B263R	pB263R	102322	103113	+	792	GENE	539.65	100
B66L	pB66L	103110	103310	-	201	GENE	128.642	100
G1340L	pG1340L	103316	107338	-	4023	GENE	2772.27	100
URF30	hypothetical protein	104342	104755	+	414	GENE	274.248	100

URF31	hypothetical protein	104566	104913	-	348	GENE	230.72	100
URF32	hypothetical protein	105626	105817	+	192	GENE	127.872	100
URF33	hypothetical protein	105766	106035	-	270	GENE	177.563	100
URF34	hypothetical protein	106449	106634	+	186	GENE	125.176	100
URF35	hypothetical protein	106801	106989	+	189	GENE	128.642	100
URF36	hypothetical protein	106980	107162	+	183	GENE	109.383	100
G1211R	pG1211R	107379	111014	+	3636	GENE	2544.61	100
CP123L	pCP123L	111011	111382	-	372	GENE	251.136	100
URF37	hypothetical protein	111483	111809	+	327	GENE	215.312	100
CP2475L	pCP2475L	111491	118918	-	7428	GENE	5089.63	100
CP204L	pCP204L	119022	119606	-	585	GENE	399.823	100
URF38	hypothetical protein	119591	119776	-	186	GENE	125.176	100
CP530R	pCP530R	119637	121229	+	1593	GENE	1105.89	100
CP80R	pCP80R	121232	121474	+	243	GENE	165.236	100
CP312R	pCP312R	121501	122439	+	939	GENE	637.876	100
O174L	pBA71V	122460	122984	-	525	GENE	348.591	100
O61R	pO61R	123034	123219	+	186	GENE	127.102	100
NP1450L	RNA polymerase subunit 1	123269	127621	-	4353	GENE	3015.33	100
URF39	hypothetical protein	123566	123763	+	198	GENE	136.346	100
URF40	hypothetical protein	124053	124514	+	462	GENE	311.612	100
URF41	hypothetical protein	124686	125102	+	417	GENE	273.863	100
URF42	hypothetical protein	124882	125151	-	270	GENE	183.726	100
URF43	hypothetical protein	126039	126239	+	201	GENE	133.265	100
URF44	hypothetical protein	127046	127246	+	201	GENE	134.035	100
URF45	hypothetical protein	127336	127566	-	231	GENE	159.458	100
NP419L	pNP419L	127704	128963	-	1260	GENE	874.004	100
NP868R	pNP868R	129028	131634	+	2607	GENE	1798.1	100
D250R	pD250R	131691	132443	+	753	GENE	521.546	100
D129L	pD129L	132624	133013	-	390	GENE	255.373	100
D79L	pD79L	133087	133326	-	240	GENE	150.984	100
pD339L	pD339L	133395	134414	-	1020	GENE	700.279	100

URF46	hypothetical protein	134202	134450	+	249	GENE	164.851	100
D1133L	pD1133L	134547	137948	-	3402	GENE	2353.17	100
URF47	hypothetical protein	137721	137993	+	273	GENE	180.644	100
URF48	hypothetical protein	137986	138144	-	159	GENE	100.908	100
D117L	pD117L	138179	138532	-	354	GENE	239.195	100
D205R	pD205R	138589	139206	+	618	GENE	423.705	100
D345L	pD345L	139233	140270	-	1038	GENE	726.472	100
S183	pS183	140270	140821	-	552	GENE	381.333	100
S273R	pS273R	140882	141703	+	822	GENE	577.785	100
URF49	hypothetical protein	141623	141874	-	252	GENE	167.933	100
P1192R	pP1192R	141738	145316	+	3579	GENE	2490.3	100
URF50	hypothetical protein	141977	142213	-	237	GENE	159.458	100
URF51	hypothetical protein	142347	142679	-	333	GENE	218.394	100
URF52	hypothetical protein	142880	143215	-	336	GENE	223.016	100
URF53	hypothetical protein	143553	143807	-	255	GENE	174.481	100
URF54	hypothetical protein	144118	144354	+	237	GENE	159.073	100
URF55	hypothetical protein	145113	145313	-	201	GENE	133.65	100
H359L	pH359L	145347	146426	-	1080	GENE	736.102	100
H171R	pH171R	146461	146976	+	516	GENE	350.132	100
H124R	pH124R	147016	147390	+	375	GENE	261.151	100
H339R	pH339R	147412	148431	+	1020	GENE	692.96	100
H108R	pH108R	148443	148769	+	327	GENE	223.016	100
H233R	pH233R	148729	149430	+	702	GENE	473.011	100
H240R	pH240R	149545	150267	+	723	GENE	491.5	100
R298L	pR298L	150264	151055	-	792	GENE	536.184	100
URF56	hypothetical protein	150855	151169	+	315	GENE	214.542	100
Q706L	pQ706L	151134	153254	-	2121	GENE	1461.82	100
QP509L	pQP509L	153212	154741	-	1530	GENE	1055.05	100
QP383R	pQP383R	154731	155882	+	1152	GENE	790.801	100
URF57	hypothetical protein	154973	155185	-	213	GENE	144.821	100
URF58	hypothetical protein	155606	155830	-	225	GENE	144.436	100

E184L	pE184L	155830	156384	-	555	GENE	376.711	100
E183L	pE183L	156428	156979	-	552	GENE	376.326	100
E423R	pE423R	157010	158281	+	1272	GENE	877.856	100
E301R	pE301R	158434	159339	+	906	GENE	618.616	100
E146L	pE146L	159373	159813	-	441	GENE	303.138	100
URF59	hypothetical protein	159632	159823	+	192	GENE	125.946	100
E199L	E199L	159997	160596	-	600	GENE	410.223	100
E165R	pE165R	160624	161121	+	498	GENE	344.354	100
E248R	pE248R	161144	161890	+	747	GENE	501.516	100
URF60	hypothetical protein	161895	162122	-	228	GENE	153.68	100
pE120R	pE120R	161932	162294	+	363	GENE	242.276	100
E296R	pE296R	162324	163214	+	891	GENE	615.535	100
E111R	pE111R	163217	163552	+	336	GENE	225.328	100
E66L	pE66L	163629	163829	-	201	GENE	132.494	100
I267L	pI267L	163777	164580	-	804	GENE	552.747	100
I226R	pI226R	164732	165412	+	681	GENE	461.84	100
I243L	pI243L	165409	166140	-	732	GENE	503.056	100
I73R	pI73R	166225	166446	+	222	GENE	146.362	100
I329L	pI329L	166682	167671	-	990	GENE	671.003	100
URF61	hypothetical protein	167487	167723	+	237	GENE	149.058	100
I215L	pI215L	167980	168627	-	648	GENE	438.343	100
I177L	pI177L	168665	169198	-	534	GENE	357.451	100
I196L	pI196L	169191	169781	-	591	GENE	385.956	100
DP238L	pDP238L	169875	170591	-	717	GENE	473.396	100
URF62	hypothetical protein	170768	171160	+	393	GENE	273.478	100
URF63	hypothetical protein	171018	171332	-	315	GENE	210.305	100
MGF 360-16R	pMGF 360-16R	171073	171822	+	750	GENE	514.998	100
DP63R	pDP63R	171604	171822	+	219	GENE	153.295	100
MGF 505-11L	pMGF 505-11L	171900	173528	-	1629	GENE	1108.21	100
MGF 100-2L	pMGF 100-2L	173647	174072	-	426	GENE	291.197	100
MGF 100-3L	pMGF 100-3L	174291	174731	-	441	GENE	294.664	100

I7L	pI7L	174829	175137	-	309	GENE	213.772	100
I8L	pI8L	175357	175668	-	312	GENE	211.846	100
URF64	hypothetical protein	175776	175898	+	123	GENE	80.8777	100
I9R	pI9R	175861	176157	+	297	GENE	207.223	100
I10L	pI10L	176230	176742	-	513	GENE	355.525	100
L11L	pL11L	176979	177212	-	234	GENE	154.836	100
MGF 360-18R	pMGF 360-18R	177444	178208	+	765	GENE	516.924	100
URF65	hypothetical protein	178076	178408	-	333	GENE	209.534	100
URF66	hypothetical protein	178096	178335	-	240	GENE	152.14	100
DP96R	pDP96R	178504	178794	+	291	GENE	199.904	100
MGF 360-19R	pMGF 360-19R	179407	180498	+	1092	GENE	748.814	100
MGF 360-20R	pMGF 360-20R	181163	181291	+	129	GENE	87.0409	100
DP60R	pDP60R	181375	181557	+	183	GENE	120.168	100

Table S5. Genetic differences between Sardinian viruses with LO2018 Minor allele

Gene Name	Product	Start	Stop	+/-	Size	Genetype	Score	% Identity
KP93L	pKP93L	71	160	-	90	GENE	65.4698	100
URF1	hypothetical protein	178	546	-	369	GENE	249.98	100
MGF 360-1L	pMGF 360-1L	655	1260	-	606	GENE	417.927	100
MGF 360-2L	pMGF 360-2L	1344	2432	-	1089	GENE	743.036	100
KP177R	pKP177R	2582	3115	+	534	GENE	370.548	100
L83L	pL83L	3253	3504	-	252	GENE	173.711	100
L60L	pL60L	3599	3802	-	204	GENE	130.568	100
MGF 360-3L	pMGF 360-3L	3987	4790	-	804	GENE	544.273	100
URF2	hypothetical protein	4729	5058	-	330	GENE	228.794	100
MGF 110-1L	pMGF 110-1L	5285	6097	-	813	GENE	564.688	100
URF3	hypothetical protein	5774	6070	+	297	GENE	199.519	100
MGF 110-13L	pMGF 110-13L	6260	6730	-	471	GENE	330.487	100
MGF 110-11L	pMGF 110-11L	6700	7065	-	366	GENE	253.447	100

MGF 110-12L	pMGF 110-12L	7254	7565	-	312	GENE	209.92	100
MGF 110-13L_1	pMGF 110-13L	7643	8140	-	498	GENE	336.65	99.4
LIS117	pLIS117	7643	7996	-	354	GENE	239.195	99.1
MGF 110-14L	pMGF 110-14L	8019	8468	-	450	GENE	313.153	100
MGF 360-4L	pMGF 360-4L	8792	9787	-	996	GENE	682.945	100
MGF 360-6L	pMGF 360-6L	10612	11739	-	1128	GENE	772.696	100
X69R	pX69R	12623	12832	+	210	GENE	140.584	100
MGF 300-1L	pMGF 300-1L	13019	13825	-	807	GENE	555.829	100
X64R	pX64R	14770	14964	+	195	GENE	132.88	100
MGF 300-2R	pMGF 300-2R	15545	16027	+	483	GENE	321.242	100
URF4	hypothetical protein	15919	16113	-	195	GENE	130.954	100
MGF 300-4L	pMGF 300-4L	16118	17104	-	987	GENE	681.019	100
MGF 300-3L	pMGF 300-3L	16118	16432	-	315	GENE	218.009	100
MGF 360-8L	pMGF 360-8L	17222	18181	-	960	GENE	665.226	100
URF5	hypothetical protein	18116	18373	+	258	GENE	172.94	100
MGF 360-9L	pMGF 360-9L	18343	19395	-	1053	GENE	733.406	100
MGF 360-10L	pMGF 360-10L	19503	20573	-	1071	GENE	731.865	100
MGF 360-11L	pMGF 360-11L	20599	21660	-	1062	GENE	733.791	100
URF6	hypothetical protein	20734	21039	+	306	GENE	203.756	100
URF7	hypothetical protein	21152	21400	+	249	GENE	154.451	100
MGF 505-1R	pMGF 505-1R	21867	23462	+	1596	GENE	1086.25	99.8
URF8	hypothetical protein	22742	22957	+	216	GENE	149.443	100
URF9	hypothetical protein	23043	23234	-	192	GENE	126.716	100
MGF 360-12L	pMGF 360-12L	23512	24564	-	1053	GENE	718.383	99.7
URF10	hypothetical protein	23978	24196	+	219	GENE	139.043	100
MGF 360-13L	pMGF 360-13L	24736	25797	-	1062	GENE	722.235	100
URF11	hypothetical protein	25969	26253	-	285	GENE	194.897	100
URF12	hypothetical protein	26281	26490	+	210	GENE	139.428	98.6
MGF 360-14L	MGF 360-14L	25969	27042	-	1074	GENE	495.352	66.4
MGF 505-2R	pMGF 505-2R	27248	28828	+	1581	GENE	1073.92	100

URF13	hypothetical protein	27808	27996	-	189	GENE	124.02	100
MGF 505-3R	pMGF 505-3R	28915	29757	+	843	GENE	576.244	100
URF14	hypothetical protein	29364	29558	-	195	GENE	131.724	100
MGF 505-4R	pMGF 505-4R	29922	31439	+	1518	GENE	1031.55	100
MGF 505-5R	pMGF 505-5R	31468	32964	+	1497	GENE	1016.53	100
MGF 505-6R	pMGF 505-6R	33162	34748	+	1587	GENE	1077.39	100
MGF 505-7R	pMGF 505-7R	34869	36275	+	1407	GENE	960.288	100
URF15	hypothetical protein	35895	36248	-	354	GENE	238.81	100
MGF 505-9R	pMGF 505-9R	36763	38283	+	1521	GENE	1045.42	100
MGF 505-10R	pMGF 505-10R	38607	40235	+	1629	GENE	1115.52	100
A224L	pA224L	40351	41025	-	675	GENE	467.233	100
A104R	pA104R	41387	41701	+	315	GENE	208.764	100
A240L	pA240L	41698	42420	-	723	GENE	495.352	100
A118R	pA118R	42332	42688	+	357	GENE	239.195	100
A151R	pA151R	42718	43173	+	456	GENE	313.538	100
MGF 360-15R	pMGF 360-15R	43518	44348	+	831	GENE	576.244	100
A238L	pA238L	44554	45270	-	717	GENE	495.352	100
URF16	hypothetical protein	45101	45313	+	213	GENE	137.887	100
URF17	hypothetical protein	45208	45420	+	213	GENE	142.895	100
URF18	hypothetical protein	45240	45449	-	210	GENE	141.739	100
A859L	pA859L	45310	47889	-	2580	GENE	1795.4	100
A179L	A179L	47891	48430	-	540	GENE	370.933	100
A137R	pA137R	48674	49087	+	414	GENE	284.648	100
F317L	pF317L	49120	50073	-	954	GENE	660.988	100
F334L	pF334L	50098	51102	-	1005	GENE	693.73	100
F778R	F778R	51123	53459	+	2337	GENE	1627.45	100
URF19	hypothetical protein	53346	53540	-	195	GENE	129.028	100
F165R	pF165R	53492	53989	+	498	GENE	344.739	100
F1055L	pF1055L	53976	57143	-	3168	GENE	2199.86	100
K205R	pK205R	57312	57929	+	618	GENE	420.624	100
BA71V-K78R	pK78R	58018	58254	+	237	GENE	152.525	100

K196R	pK196R	58251	58841	+	591	GENE	407.142	100
K145R	pK145R	58857	59294	+	438	GENE	292.738	100
K421R	pK421R	59327	60592	+	1266	GENE	869.381	100
EP1242L	pEP1242L	60611	64339	-	3729	GENE	2596.62	100
URF20	hypothetical protein	64037	64360	+	324	GENE	218.394	100
EP84R	EP84R	64418	64672	+	255	GENE	159.844	100
EP424R	pEP424R	64708	65982	+	1275	GENE	881.322	100
EP152R	pEP152R	66465	66923	+	459	GENE	317.005	100
EP153R	pEP153R	66926	67387	+	462	GENE	302.368	100
EP402R	pEP402R	67487	68671	+	1185	GENE	758.444	100
URF21	hypothetical protein	68211	68657	+	447	GENE	269.24	100
EP364R	pEP364R	68780	69874	+	1095	GENE	758.444	99.7
M1249L	pM1249L	69938	73261	-	3324	GENE	2308.49	100
M448R	pM448R	73727	75073	+	1347	GENE	936.406	100
C129R	pC129R	75160	75549	+	390	GENE	267.314	100
C84L	pC84L	75596	75850	-	255	GENE	168.703	100
C717R	pC717R	76274	78427	+	2154	GENE	1491.09	100
URF22	hypothetical protein	78411	78644	-	234	GENE	157.147	100
C122R	pC122R	78414	78731	+	318	GENE	217.238	100
C257L	pC257L	79130	79903	-	774	GENE	533.872	100
C475L	pC475L	79882	81309	-	1428	GENE	982.245	100
C315R	pC315R	81362	82309	+	948	GENE	649.432	100
C147L	pC147L	82377	82820	-	444	GENE	296.59	100
URF23	hypothetical protein	82453	83205	+	753	GENE	503.827	100
URF24	hypothetical protein	82837	83205	+	369	GENE	255.758	100
C62L	pC62L	82980	83168	-	189	GENE	124.79	100
C962R	pC962R	83250	86138	+	2889	GENE	2007.26	100
C962R_1	pC962R_1	84129	86138	+	2010	GENE	1403.27	100
URF25	hypothetical protein	86089	86247	-	159	GENE	105.531	100
URF26	hypothetical protein	86272	86487	-	216	GENE	147.132	100
B962L	pB962L	86276	89164	-	2889	GENE	2001.48	100

B119L	pB119L	89124	89483	-	360	GENE	247.669	100
URF27	hypothetical protein	89389	89658	+	270	GENE	182.185	100
B318L	pB318L	89464	90420	-	957	GENE	664.455	100
B438L	pB438L	90387	91703	-	1317	GENE	905.975	100
B169L	pB169L	91716	92225	-	510	GENE	340.117	100
B475L	pB475L	92242	93669	-	1428	GENE	958.362	100
B354L	pB354L	93806	94870	-	1065	GENE	736.102	100
B602L	pB602L	94881	96497	-	1617	GENE	1116.68	100
URF28	hypothetical protein	95827	96033	+	207	GENE	123.635	100
URF29	hypothetical protein	96311	96520	-	210	GENE	139.813	100
B385R	pB385R	96552	97709	+	1158	GENE	801.201	100
B646L	pB646L	97807	99747	-	1941	GENE	1347.41	100
B125R	pB125R	99787	100164	+	378	GENE	255.373	100
B117L	pB117L	100137	100490	-	354	GENE	244.202	100
B407L	pB407L	100497	101720	-	1224	GENE	837.41	100
B175L	pB175L	101748	102275	-	528	GENE	365.54	100
B263R	pB263R	102322	103113	+	792	GENE	539.65	100
B66L	pB66L	103110	103310	-	201	GENE	128.642	100
G1340L	pG1340L	103316	107338	-	4023	GENE	2770.34	99.9
URF30	hypothetical protein	104342	104755	+	414	GENE	274.248	100
URF31	hypothetical protein	104566	104913	-	348	GENE	230.72	100
URF32	hypothetical protein	105626	105817	+	192	GENE	127.872	100
URF34	hypothetical protein	106449	106634	+	186	GENE	125.176	100
URF35	hypothetical protein	106801	106989	+	189	GENE	128.642	100
URF36	hypothetical protein	106980	107162	+	183	GENE	109.383	100
G1211R	pG1211R	107379	111014	+	3636	GENE	2544.61	100
CP123L	pCP123L	111011	111382	-	372	GENE	251.136	100
URF37	hypothetical protein	111483	111809	+	327	GENE	215.312	100
CP2475L	pCP2475L	111491	118918	-	7428	GENE	5086.16	100
CP204L	pCP204L	119022	119606	-	585	GENE	399.823	100
URF38	hypothetical protein	119591	119776	-	186	GENE	125.176	100

CP530R	pCP530R	119637	121229	+	1593	GENE	1105.89	100
CP80R	pCP80R	121232	121474	+	243	GENE	165.236	100
CP312R	pCP312R	121501	122439	+	939	GENE	637.876	100
O174L	pBA71V	122460	122984	-	525	GENE	348.591	100
O61R	pO61R	123034	123219	+	186	GENE	127.102	100
NP1450L	RNA polymerase subunit 1	123269	127621	-	4353	GENE	3015.33	100
URF39	hypothetical protein	123566	123763	+	198	GENE	136.346	100
URF40	hypothetical protein	124053	124514	+	462	GENE	311.612	100
URF41	hypothetical protein	124686	125102	+	417	GENE	273.863	100
URF42	hypothetical protein	124882	125151	-	270	GENE	183.726	100
URF43	hypothetical protein	126039	126239	+	201	GENE	133.265	100
URF44	hypothetical protein	127046	127246	+	201	GENE	134.035	100
URF45	hypothetical protein	127336	127566	-	231	GENE	159.458	100
NP419L	pNP419L	127704	128963	-	1260	GENE	874.004	100
NP868R	pNP868R	129028	131634	+	2607	GENE	1798.1	100
D250R	pD250R	131691	132443	+	753	GENE	521.546	100
D129L	pD129L	132624	133013	-	390	GENE	255.373	100
D79L	pD79L	133087	133326	-	240	GENE	150.984	100
pD339L	pD339L	133395	134414	-	1020	GENE	700.279	100
URF46	hypothetical protein	134202	134450	+	249	GENE	164.851	100
D1133L	pD1133L	134547	137948	-	3402	GENE	2353.17	100
URF47	hypothetical protein	137721	137993	+	273	GENE	180.644	100
URF48	hypothetical protein	137986	138144	-	159	GENE	100.908	100
D117L	pD117L	138179	138532	-	354	GENE	239.195	100
D205R	pD205R	138589	139206	+	618	GENE	423.705	100
D345L	pD345L	139233	140270	-	1038	GENE	726.472	100
S183	pS183	140270	140821	-	552	GENE	381.333	100
S273R	pS273R	140882	141703	+	822	GENE	577.785	100
URF49	hypothetical protein	141623	141874	-	252	GENE	167.933	100
P1192R	pP1192R	141738	145316	+	3579	GENE	2490.3	100
URF50	hypothetical protein	141977	142213	-	237	GENE	159.458	100

URF51	hypothetical protein	142347	142679	-	333	GENE	218.394	100
URF52	hypothetical protein	142880	143215	-	336	GENE	223.016	100
URF53	hypothetical protein	143553	143807	-	255	GENE	174.481	100
URF54	hypothetical protein	144118	144354	+	237	GENE	159.073	100
URF55	hypothetical protein	145113	145313	-	201	GENE	133.65	100
H359L	pH359L	145347	146426	-	1080	GENE	736.102	100
H171R	pH171R	146461	146976	+	516	GENE	350.132	100
H124R	pH124R	147016	147390	+	375	GENE	261.151	100
H339R	pH339R	147412	148431	+	1020	GENE	692.96	100
H108R	pH108R	148443	148769	+	327	GENE	223.016	100
H233R	pH233R	148729	149430	+	702	GENE	473.011	100
H240R	pH240R	149545	150267	+	723	GENE	491.5	100
R298L	pR298L	150264	151055	-	792	GENE	536.184	100
URF56	hypothetical protein	150855	151169	+	315	GENE	214.542	100
Q706L	pQ706L	151134	153254	-	2121	GENE	1461.82	100
QP509L	pQP509L	153212	154741	-	1530	GENE	1055.05	100
QP383R	pQP383R	154731	155882	+	1152	GENE	790.801	100
URF57	hypothetical protein	154973	155185	-	213	GENE	144.821	100
URF58	hypothetical protein	155606	155830	-	225	GENE	144.436	100
E184L	pE184L	155830	156384	-	555	GENE	376.711	100
E183L	pE183L	156428	156979	-	552	GENE	376.326	100
E423R	pE423R	157010	158281	+	1272	GENE	877.856	100
E301R	pE301R	158434	159339	+	906	GENE	618.616	100
E146L	pE146L	159373	159813	-	441	GENE	303.138	100
URF59	hypothetical protein	159632	159823	+	192	GENE	125.946	100
E199L	E199L	159997	160596	-	600	GENE	410.223	100
E165R	pE165R	160624	161121	+	498	GENE	344.354	100
E248R	pE248R	161144	161890	+	747	GENE	501.516	100
URF60	hypothetical protein	161895	162122	-	228	GENE	153.68	100
pE120R	pE120R	161932	162294	+	363	GENE	242.276	100
E296R	pE296R	162324	163214	+	891	GENE	615.535	100

E111R	pE111R	163217	163552	+	336	GENE	225.328	100
E66L	pE66L	163629	163829	-	201	GENE	132.494	100
I267L	pI267L	163777	164580	-	804	GENE	552.747	100
I226R	pI226R	164732	165412	+	681	GENE	461.84	100
I243L	pI243L	165409	166140	-	732	GENE	503.056	100
I73R	pI73R	166225	166446	+	222	GENE	146.362	100
I329L	pI329L	166682	167671	-	990	GENE	671.003	100
URF61	hypothetical protein	167487	167723	+	237	GENE	149.058	100
I215L	pI215L	167980	168627	-	648	GENE	438.343	100
I177L	pI177L	168665	169198	-	534	GENE	357.451	100
I196L	pI196L	169191	169781	-	591	GENE	385.956	100
DP238L	pDP238L	169875	170591	-	717	GENE	473.396	100
URF62	hypothetical protein	170768	171160	+	393	GENE	273.478	100
URF63	hypothetical protein	171018	171332	-	315	GENE	210.305	100
MGF 360-16R	pMGF 360-16R	171073	171822	+	750	GENE	514.998	100
DP63R	pDP63R	171604	171822	+	219	GENE	153.295	100
MGF 505-11L	pMGF 505-11L	171900	173528	-	1629	GENE	1108.21	100
MGF 100-2L	pMGF 100-2L	173647	174072	-	426	GENE	291.197	100
MGF 100-3L	pMGF 100-3L	174291	174731	-	441	GENE	294.664	100
I7L	pI7L	174829	175137	-	309	GENE	213.772	100
I8L	pI8L	175357	175668	-	312	GENE	211.846	100
URF64	hypothetical protein	175776	175898	+	123	GENE	80.8777	100
I9R	pI9R	175861	176157	+	297	GENE	207.223	100
I10L	pI10L	176230	176742	-	513	GENE	355.525	100
L11L	pL11L	176979	177212	-	234	GENE	154.836	100
MGF 360-18R	pMGF 360-18R	177444	178208	+	765	GENE	516.924	100
URF65	hypothetical protein	178076	178408	-	333	GENE	209.534	100
URF66	hypothetical protein	178096	178335	-	240	GENE	152.14	100
DP96R	pDP96R	178504	178794	+	291	GENE	197.593	99
MGF 360-19R	pMGF 360-19R	179407	180498	+	1092	GENE	748.814	100
MGF 360-20R	pMGF 360-20R	181163	181291	+	129	GENE	87.0409	100

DP60R	pDP60R	181375	181557	+	183	GENE	120.168	100
